# Activated FGFR2 signalling as a biomarker for selection of intrahepatic cholangiocarcinoma patients candidate to FGFR targeted therapies

**DOI:** 10.1038/s41598-024-52991-8

**Published:** 2024-02-07

**Authors:** Giovanni Brandi, Valeria Relli, Marzia Deserti, Andrea Palloni, Valentina Indio, Annalisa Astolfi, Salvatore Serravalle, Alessandro Mattiaccio, Francesco Vasuri, Deborah Malvi, Chiara Deiana, Maria Abbondanza Pantaleo, Matteo Cescon, Alessandro Rizzo, Masaru Katoh, Simona Tavolari

**Affiliations:** 1 Medical Oncology, IRCCS Azienda Ospedaliero-Universitaria di Bologna, Bologna, Italy; 2 Department of Medical and Surgical Sciences, University of Bologna, Bologna, Italy; 3 Center for Applied Biomedical Research, University of Bologna, Bologna, Italy; 4 Department of Veterinary Medical Sciences, University of Bologna, Bologna, Italy; 5 IRCCS Azienda Ospedaliero-Universitaria di Bologna, Bologna, Italy; 6 Division of Pediatrics, IRCCS-Azienda Ospedaliero-Universitaria of Bologna, Bologna, Italy; 7 Pathology Unit, IRCCS Azienda Ospedaliero-Universitaria of Bologna, Bologna, Italy; 8 General Surgery and Transplant Unit, IRCCS Azienda Ospedaliero-Universitaria di Bologna, Bologna, Italy; 9 IRCCS Istituto Tumori “Giovanni Paolo II” of Bari, Bari, Italy; 10 M & M Precision Medicine, Tokyo, Japan; 11 Department of Omics Network, National Cancer Center, Tokyo, Japan

**Keywords:** Cancer, Medical research, Oncology

## Abstract

FGFR inhibitors have been developed to inhibit FGFR activation and signal transduction; notwithstanding, currently the selection of intrahepatic cholangiocarcinoma (iCCA) patients for these drugs only relies on the detection of *FGFR2* genetic alterations (GAs) in tumor tissues or circulating tumor DNAs, without concomitant assessment of FGFR2 signalling status. Accordingly, we performed multi-omic analyses of *FGFR2* genes and FGFR2 signalling molecules in the tissue samples from 36 iCCA naïve patients. Gain-of-function *FGFR2* GAs were detected in 7 patients, including missense mutations (n = 3; p.F276C, p.C382R and p.Y375C), translocations (n = 1) and copy number gain (n = 4; CNV ≥ 4). In contrast, among 29 patients with wild-type *FGFR2*, 4 cases showed activation of FGFR2 signalling, as they expressed the FGFR2 ligand FGF10 and phosphorylated FGFR2/FRS2α proteins; the remaining 25 cases resulted negative for activated FGFR2 signalling, as they lacked FGFR2 (n = 8) or phosphorylated FRS2α (n = 17) expression. Overall, we found that activation of FGFR2 signalling occurs not only in iCCA naïve patients with *FGFR2* GAs, but also in a subgroup carrying wild-type *FGFR2*. This last finding entails that also this setting of patients could benefit from FGFR targeted therapies, widening indication of these drugs for iCCA patients beyond current approval. Future clinical studies are therefore encouraged to confirm this hypothesis.

## Introduction

Intrahepatic cholangiocarcinoma (iCCA) is an aggressive cancer of the bile ducts within the liver whose incidence has been rising in the last forty years, especially in Western countries^1^.

In patients with locally advanced, unresectable or metastatic disease, currently the majority of the diagnosed iCCA cases, gemcitabine plus cisplatin has been the mainstay in first-line setting for more than a decade^2^. Recently, basing on the interim analysis from the TOPAZ-1 trial, first-line treatment with the PD-L1 inhibitor Durvalumab in combination with gemcitabine and cisplatin has been approved by Food and Drug Administration (FDA) in patients with advanced disease^3^; in addition, 5-fluorouracil/oxaliplatin chemotherapy and liposomal irinotecan plus fluorouracil and leucovorin have been suggested as second-line chemotherapy according to ABC-06 and NIFTY trials^4,5^. Despite these therapeutic advances, the prognosis of iCCA remains poor, with a 5-year survival rate from 5 to 10% for patients with unresectable disease^6^, and from 20 to 35% in patients undergone curative-intent surgery^7^.

In this scenario, considerable efforts are ongoing worldwide to identify actionable targets that can guide treatment decision and improve the survival of iCCA patients; interestingly, actionable molecular alterations are detected in over 40% of iCCA cases^8^. Among actionable targets, isocitrate dehydrogenase (IDH) 1 and 2 and fibroblast growth factor receptor (FGFR) 2 alterations have gained increased clinical interest and recently the FGFR inhibitors Pemigatinib, Infigratinib and Futibatinib have been approved as the first targeted therapies in advanced iCCA patients with FGFR2 fusions or rearrangements^9–11^.

FGFR2 is a tyrosine kinase receptor with a core structure containing an extracellular ligand binding domain (ECD), a hydrophobic transmembrane domain (TD) and an intracellular tyrosine kinase domain (TKD)^12^. Ligand-dependent receptor dimerization results in TKD phosphorylation, that in turn phosphorylates the FGFR substrate 2α (FRS2α), a key adaptor protein that initiates FGFR downstream signalling inducing the activation of mitogen activated protein kinase (MAPK) and the phosphoinositide-3-kinase (PI3K)/AKT oncogenic pathways^13^.

Aberrant FGFR signalling is frequently observed in many cancer types as a result of *FGFR* gene amplifications, point mutations, chromosomal translocations/fusions, and abnormal autocrine/paracrine release of the FGF ligands by stromal/cancer cells^14^. In iCCA, most of detected *FGFR* genomic alterations (GAs) are *FGFR2* activating fusions/rearrangements (up to 15% of cases); activating *FGFR2* amplifications and mutations also occurs in this disease, even though with a lower frequency than fusions/rearrangements^15–18^. Recent clinical studies have provided evidences that the response to FGFR inhibitors differs among iCCA patients, based on the presence and type of the *FGFR* GAs detected in the tumor tissue. Patients with *FGFR2* amplifications or point mutations are usually less responsive than patients with fusions/rearrangements; moreover, patients with no *FGFR* GAs have shown no clinical response to these drugs^11,19–22^. However it is worth to underline that, although FGFR targeted therapies have been specifically developed to inhibit FGFR activation and downstream signalling cascade, currently in clinical practice the selection of iCCA patients candidate to these treatments only relies on the detection of *FGFR2* GAs in the tumor tissue, without any information about the status of FGFR downstream signalling cascade.

To address this issue, here we performed concomitant molecular characterization of FGFR2 and FGFR2 downstream signalling cascade in the tissue samples from 36 iCCA naïve patients, in order to identify additional subpopulations of patients that are outside of currently approved indications but could potentially benefit from FGFR inhibitors.

## Methods

### Study population

A total of 36 consecutive iCCA naïve patients who underwent surgery with curative intent at IRCCS Azienda Ospedaliero-Universitaria of Bologna (Bologna, Italy) from May 2014 to December 2017 were included in the study according to the following inclusion criteria: (a) patients aged ≥ 18 with confirmed diagnosis of iCCA; (b) patients not receiving any chemotherapy and/or radiotherapy regimens in the last 5 years prior to surgery; (c) presence of an adequate amount of tumor tissue for molecular analysis. Morphological classification of the tumors was based on the WHO criteria^23^. Tumors were graded according to the international tumor-node-metastasis (TNM) system^24^.

The study was approved by local Ethics Committee (EUDRACT 211201UV/Tess) and conducted according to the principles of Declaration of Helsinki; written informed consent to participate in the study was obtained from all participants.

### Study flow chart

**Figure 1:**
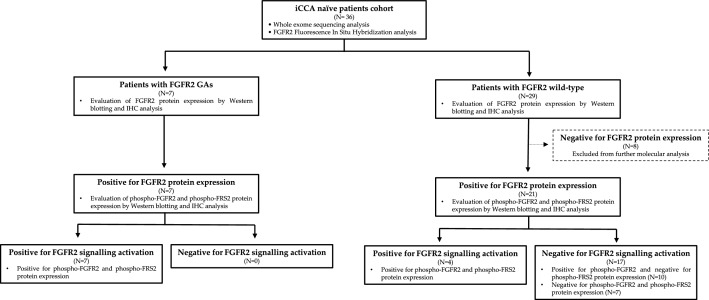
Study flow chart for the assessment of FGFR2 status and FGFR2 signalling activation in the study population.

The study flow chart is summarized in Fig. 1. Briefly, a total of 36 iCCA naïve patients meeting inclusion criteria were enrolled. For each patient, the occurrence of *FGFR2* mutations, rearrangements/fusions and copy number variations (CNVs) was assessed by whole exome sequencing and fluorescence in situ hybridization analysis. Based on *FGFR2* molecular status, patients were then assigned to two subgroups: patients with *FGFR2* GAs and patients with wild-type *FGFR2*. In both subgroups, FGFR2 protein expression in tumor tissue was assessed by Western blotting and immunohistochemistry analysis. Patients resulting negative for FGFR2 expression were excluded from further molecular analyses, while patients positive for FGFR2 expression were assessed for FGFR2 signalling cascade activation by evaluation of phospho-FGFR2 and phospho-FRS2α protein expression. Finally, patients resulting positive for the expression of these proteins were classified as “positive for FGFR2 signalling activation”, whereas those negative for such expression as “negative for FGFR2 signalling activation”.

### Whole exome sequencing (WES)

Fresh tumor tissue and peripheral blood samples were collected during surgery from each patient. Tumor specimens were evaluated by two expert pathologists (V.F. and M.D.) to ensure the presence at least 20% of cancer cells. DNA was extracted from primary tumor samples with a DNA mini-kit (Qiagen, Milan, Italy), following manufacturer’s instructions. WES was performed on HiScanSQ platform in accordance with Nextera Rapid Exome Enrichment protocol (Illumina, San Diego, California, USA). Briefly, 100 ng of genomic DNA was tagged and fragmented by the Nextera transposome. The Nextera transposome simultaneously fragments the genomic DNA and adds adapter sequences to the ends. The products were then amplified and exome regions were enriched. The enriched libraries were amplified by PCR and quantified using PicoGreen assay (Life Technologies, Milan, Italy). Paired-end libraries were sequenced at 2 × 100 bp read length using Illumina Sequencing by synthesis (SBS) technology.

### Bioinformatics analysis

Raw data, achieved with Illumina platform, were firstly processed with Illumina Bcltofastq function software, in order to convert the data in human-readable FASTQ format, containing the paired-end reads and the read quality information. After adapter removal and trimming based on quality score (https://adapterremoval.readthedocs.io), the paired-end reads were mapped on the human reference genome hg19 with BWA alignment tool (http://bio-bwa.sourceforge.net). The alignments were processed with samtools (http://samtools.sourceforge.net) to remove PCR duplicates and with GATK (https://gatk.broadinstitute.org/) in order to perform local realignment around the insertions and deletions (Indels) and base quality score recalibration. GATK was also adopted to call Indels (with HaplotypeCaller function) while single nucleotide variants (SNV) were detected with MuTect (https://software.broadinstitute.org/cancer/cga/mutect). Variants included on dbSNP, 1000Genomes, EVS and Exac human variability databases with frequency greater than 1% were excluded. All variants from the matched blood-tumor pairs that were unique in the tumor sample will be called Somatic. The functional effect of coding variants was predicted with SnpEff (http://snpeff.sourceforge.net).

### Clinical actionability of somatic coding variants

Clinical actionability of somatic coding variants was referred to the expert-guided precision oncology knowledge database OncoKB (https://oncokb.org/)^25^. Genomic alterations were classified as actionable using a level of evidence scale from 1 to 4, as follows: (a) level 1: treatment approved by FDA; (b) level 2: standard of care; (c) level 3: clinical evidence; (d) level 4: biological evidence^25^.

### Sanger sequencing

PCR amplification of *FGFR2* exon 7 was performed under standard conditions and purified PCR products were sequenced on both strands with the Big Dye Terminator v1.1 Cycle Sequencing kit (Applied Biosystems, Monza, Italy). The reaction products were analysed on the ABI 3730 Genetic Analyzer (Life Technologies, CA, USA). Specific primer pairs (FGFR2_7F-AGAATGGTCGTCGCCTTTTG and FGFR2_7R-AATCACTCGCACATGGAAGC; FGFR2_7F-GGGGCCACAGTGTTATTTCA and FGFR2_7R-CTCTCTGCTGGCTAGTCAAAA) were designed with Primer Express 3.0 software (Applied Biosystem).

### Multiple sequence alignment (MSA)

MSA was performed by the Clustal Omega online tool (https://www.ebi.ac.uk/Tools/msa/clustalo/) that uses seeded guide trees and HMM profile-profile techniques to generate alignments between sequences. Human FGFR2 protein was aligned against orthologous proteins from five phylogenetically selected species.

### Fluorescence in situ hybridization (FISH)

FISH analysis was performed on formalin-fixed, paraffin-embedded (FFPE) iCCA tissue samples pre-treated with the Paraffin Pretreatment Reagent Kit (Abbott Molecular Inc., Germany), following the manufacturer’s protocol. Two Empire Genomics probes (Empire Genomics, Buffalo, NY) were employed following the manufacturer’s protocol. Specifically, the *FGFR2* gene probe (orange) maps on Chromosome 10q26.13 and the *CEP10* probe maps to the centromeric region of Chromosome 10. Tumour cells were counterstained with 40,6-diamidino-2-phenylindole (DAPI) for nuclear detection. Analysis was performed using an Olympus BX53 microscopy equipped with the appropriate filter sets and CytoVision software (Leica Biosystems, Nussloch, Germany). Ratio of *FGFR2* signals to *CEP10* signals (*FGFR2/CEP10* ratio) > 2 was interpreted as gene amplification, as previously reported^26^.

### Cell line

The human non-malignant cholangiocyte H69 cell line was a generous gift of Prof. Jesus Banales (Department of Liver and Gastrointestinal Diseases, Donostia University Hospital, Donostia-San Sebastian, Spain). Cells were cultured in DMEM and DMEM/Ham’s F12 supplemented with 10% fetal bovine serum, penicillin/streptomycin (all from Euroclone, Milan, Italy), 1.8 × 10^–4^ M adenine, 5 μg/ml insulin, 5.5 × 10^–6^ M epinephrine, 2 × 10^–9^ M triiodothyronine, 1.64 × 10^–6^ M epidermal growth factor and 1.1 × 10^–6^ M hydrocortisone (all from Sigma-Aldrich, St. Louis, MO).

### Immunohistochemistry (IHC)

IHC on FFPE iCCA tissue samples was carried out using Novolink Polymer Detection System (Leica Mycrosystems, Germany), as previously reported^27^. Tumor sections were incubated overnight at 4 °C with FGFR2 (Abcam, Cambridge, UK), phospho-FGFR2 (Tyr769) (Invitrogen, MA, USA), phospho-FRS2α (Tyr436) and FGF10 (both from R&D Systems, Minneapolis, USA) primary antibodies. FGFR2 expression was scored according to cytoplasmic staining intensity (0: negative; 1 + : weak; 2 + : moderate; 3 + : high) and the proportion of positive stained cells, as previously reported^28^. Cases were considered positive for FGFR2 expression when staining intensity was moderate/strong (score ≥ 2 +) and the proportion of positively stained cells > 10%; a staining intensity negative/weak (score ≤ 1 +) was considered as negative for FGFR2 expression. Cell membrane positivity was assessed as present (≥ 10% of positive tumor cells) or absent. FGF10 ligand, phosphorylated FGFR2 and phosphorylated FRS2α immunoreactivity was semi-quantitatively assessed and graded on the basis of the number of tumor positive cells as follows: 0 (no positive cells), 1 + (1–25% positive cells), 2 + (25–50% positive cells) and 3 + (> 50% positive cells)^22^. Mismatch repair (MMR) protein expression (MLH1, MSH2, MSH6 and PMS2) was assessed by VENTANA MMR IHC Panel (Ventana Medical Systems, Inc.; Roche Diagnostics), according to the manufacturer's protocol. Primary antibodies were the following: anti-MLH1 (M1) (cat. no. 518-114336), anti-MSH2 (cat. no. G219-1129), anti-MSH6 (cat. no. SP93) and anti-PMS2 (cat. no. A16-4). MMR-deficient cases were defined by the loss of nuclear immunoreaction in at least one of the four proteins. All slides were evaluated by two expert pathologists, blind each other, with a good agreement in IHC results (data not shown); the few discordant cases were collegially discussed.

### Western blotting

Western blotting was performed as previously reported^27^ with the following primary antibodies: FGFR2 (D4L2V), phospho-FRS2α (Tyr436) and GAPDH (14C10) (Cell Signalling Technology, MA, USA), phospho-FGFR2 (pSer782) (TermoFisher, MA, USA) and FRS2α (Abcam, CB, UK). After probing for phosphorylated proteins, membranes were stripped by Re-Blot Plus Western Blot Recycling Kit (Chemicon International, Temecula, Calif) and incubated with antibodies against the corresponding total protein. GADPH was used for equal protein loading. Digital images of X-ray films were captured by ChemiDoc XRSþ (Image Lab Software, Bio-Rad).

### Statistical analysis

Analysis of WES data has been described above. Statistical comparisons were made by Student’s t test with GraphPad Prism 8 (GraphPad Software, San Diego, CA, USA). A p-value < 0.05 was considered statistically significant.

### Ethics approval and consent to participate

The study was approved by local Ethics Committee of IRCCS Azienda Ospedaliero-Universitaria di Bologna (EUDRACT 211201UV/Tess) and conducted according to the principles of Declaration of Helsinki; written informed consent to participate in the study was obtained from all participants.

## Results

### Genomic landscape of iCCA naïve patients

The demographic and clinicopathological characteristics of the 36 iCCA naïve patients included in the study are reported in Table 1. The cohort consisted of 20 men and 16 women; the median age at surgery was 61.6 years old (38–85). All patients underwent surgery with curative intent at our hospital; resection margins were R0 in 23 (64%) cases and R1 in 12 (33%) cases. According to the 8th edition of the AJCC staging system, 9 patients (2%) were classified as T1, 20 patients (56%) as T2, 2 patients (5%) as T3 and 3 patients as T4 (9%). Six patients (17%) had loco-regional lymph node metastasis. Tumors were classified as G1 in 4 cases (11%), G2 in 14 cases (39%) and G3 in 15 cases (42%). Pathological classification was stage I in 3 patients (8%), stage II in 11 (31%) and stage III in 9 cases (25%). One patient was classified as stage IV because he received a concomitant right adrenalectomy for synchronous metastasis.

**Table 1:** Baseline characteristics of the thirty-six iCCA naïve patients included in the study.

Characteristics	Total (n = 36)
Age at surgery (yr), median (range)	61.6 (38–85)
Gender, n (%)	
Female	16 (44)
Male	20 (56)
Disease stage, n (%)	
I	3 (8)
II	11 (31)
III	9 (25)
IV	1 (3)
NA	12 (33)
Size and extent (T), n (%)	
T1	9 (25)
T2	20 (56)
T3	2 (5)
T4	3 (9)
NA	2 (5)
Regional lymph nodes (N), n (%)	
N0	17 (47)
N1	6 (17)
NA	13 (36)
Distant metastases (M), n (%)	
M0	32 (89)
M1	1 (3)
NA	3 (8)
Resection margins, n (%)	
R0	23 (64)
R1	12 (33)
NA	1 (3)
Histological grade, n (%)	
G1	4 (11)
G2	14 (39)
G3	15 (42)
NA	3 (8)

The total number of somatic coding variants per tumor detected by WES analysis ranged from 1 to 41 (median = 7) across the 36 iCCA cases (the full list of variants is reported in Supplementary Fig. 1). Three cases (T12, T18 and T28) showed a hypermutated profile compared to the rest of the cohort (mean number of somatic variants = 35.3 *vs* 6.4, p-value < 0.0001) (Supplementary Fig. 1); further IHC analysis of mismatch repair (MMR) proteins showed the loss of PMS2 and MLH1 expression in case T12 and T18, and loss of MSH2 and MSH6 expression in case T28, confirming the occurrence of MMR-deficiency in these hypermutated cases (Supplementary Fig. 2).

**Figure 2:**
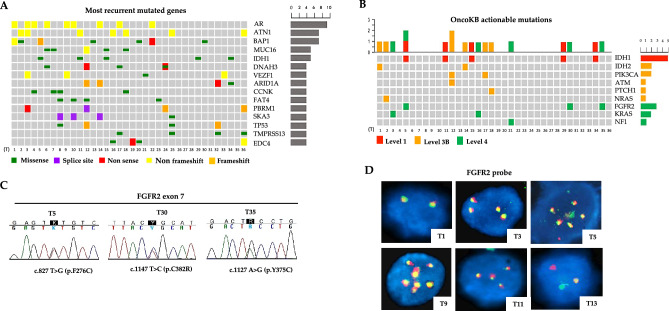
(**A**) Oncoplot of the 15 most recurrent mutated genes in the cohort of 36 naïve iCCA patients. Each number represents a patient. Green: missense mutations; purple: splice-site mutations; red: non-sense mutations; yellow: non-frameshift mutations; orange: frameshift mutations; (**B**) Somatic coding variants with a preclinical or clinical evidence of treatment according to OncoKb database in the cohort of 36 iCCA naïve patients. Each number represents a patient. Red: Level 1; orange: Level 3B; green: Level 4; (**C**) Validation by Sanger sequencing of FGFR2 c.827T>G (p.F276C), c.1147T>C (p.C382R ) and c.1127A>G (p.Y375C) missense mutations identified by WES analysis in cases T5, T30 and T35; (**D**) FGFR2 Break Apart FISH analysis showing the presence of a translocation in case T13 and FGFR2 CNV ≥ 4 in T3, T9, T11 and T5 cases. T1 was representative of cases with two FGFR2 normal signals. DAPI counterstain. Magnification 60X. T: tumor.

Most recurrent mutated genes in the whole cohort of patients were *AR* (androgen receptor, 25%), *ATN1* and *BAP1* (19.4%), *IDH1* and *MUC16* (13.8%), *ARID1A*, *CCNK*, *DNAH3*, *EDC4, FAT4*, *PBRM1*, *SKA3*, *TMPRSS13*, *TP53* and *VEZF1* (11.1%) (Fig. 2A). According to the OncoKB database, at least one somatic coding variant with a preclinical or clinical evidence of treatment was detected in 16 (44.4%) out of 36 patients (Fig. 2B), in line with previous studies reporting potentially targetable genetic variants in about 50% of CCA patients^22,29^. Overall, a total of eighteen actionable mutations were identified in these patients, including the IDH1 hot-spot mutation p.R132C with level of evidence 1, IDH2, PIK3CA, ATM, PTCH1 and NRAS mutations with level of evidence 3B and FGFR2, KRAS and NF1 mutations with level of evidence 4 (Supplementary Table 1). Of note, T5 case carried concomitant IDH1 p.R132C and FGFR2 p.F276C actionable mutations, whereas T12 case the PIK3CA p.G1049R and ATM p.A1812fs actionable mutations (Fig. 2B).

### FGFR2 genomic alterations (GAs) in iCCA naïve patients

The *FGFR2* c.827T>G (p.F276C), c.827T>G (p.C382R) and c.1127A>G (p.Y375C) somatic variants identified by WES analysis in T5, T30 and T35 cases were then confirmed by Sanger sequencing (Fig. 2C). As shown in Supplementary Fig. 3A, the p.F276C mutation occurred in the IgIII ECD of FGFR2, while p.Y375C and p.C382R mutations in the TMD of the receptor. MSA analysis revealed that F276, Y375 and C382 residues of FGFR2 were phylogenetically conserved from zebrafish to humans (Supplementary Fig. 3B), suggesting that these sites might play an essential role for proper function of FGFR2 receptor. Indeed, these FGFR2 mutations were predicted “likely gain-of-function” and “likely oncogenic” in the OncoKB database^25^.

We then assessed the occurrence of *FGFR2* fusions/rearrangements in the cohort of 36 iCCA naïve patients by FISH analysis. As shown in Fig. 2D, 31 out of 36 cases (86.1%) showed combined fluorescence signals (yellow) derived from wild-type *FGFR2* gene (T1 as a representative case), whereas one case (2.8%) showed separate fluorescence signals (red and green) as a result of *FGFR2* gene translocation (T13) and 4 cases (11.1%) showed *FGFR2* copy number gains (CNV ≥ 4) (T3, T9, T11 and T5). Further FISH analysis with CEP10 probe revealed that the increase in FGFR2 CNV in these four cases was not due to gene amplification, but to a gain of chromosome 10 (Supplementary Fig. 4).

Overall *FGFR2* GAs were detected in 7 out of 36 iCCA naïve patients (19.4%). Of note, in case T5, *FGFR2* CNV co-occurred along with FGFR2 p.F276C, BAP1 and IDH1 mutations (Fig. 2A). Similarly, the patient with the FGFR2 p.C382R variant (T30) and the patient with *FGFR2* translocation (T13) also carried a concomitant BAP1 mutation (Fig. 2A). Furthermore, in case T11, *FGFR2* CNV co-occurred with IDH1 mutation (Fig. 2A). These findings are in line with previous genomic studies reporting that *FGFR*, *BAP1* and *IDH1/2* alterations cluster in a subgroup of iCCA patients that is distinct from the subgroup of iCCA patients with *TP53*, *KRAS*, *SMAD4* and *ARID1A* alterations^8,15^.

### FGFR2 expression and signalling activation in iCCA naïve patients with FGFR2 GAs

**Figure 3:**
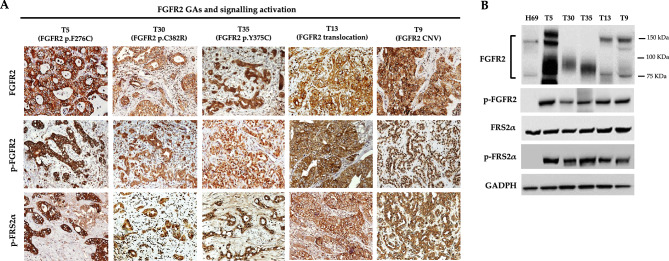
(**A**) Representative IHC images of FGFR2, phospho-FGFR2 and phospho-FRS2α protein expression in iCCA naïve patients with FGFR2 GAs. Case T5 represents the patients with FGFR2 p.F276C mutation and FGFR2 CNV > 4; cases T30 and T35 represent the patients with FGFR2 p.C382R and p.Y375C mutations, respectively; case T13 represents the patient with FGFR2 translocation and case T9 was representative for patients with increased FGFR2 copy number. Magnification 20X; (**B**) Western blotting analysis of FGFR2, phospho-FGFR2, phospho-FRS2α and FRS2α protein expression in iCCA naïve patients with FGFR2 GAs. T5, T30 and T35 cases served as refence for activated FGFR2 signalling in tumor tissue and the human cholangiocyte H69 cell line as negative control. In FGFR2 immunoblotting, the lower band from ~ 75 to ~ 100 KDa refers to the unglycosylated/partially glycosylated form of FGFR2 protein, whereas the upper band of ~ 150KDa to the fully glycosylated form of the receptor. GAPDH was used as a quantitative control for equal loading.

Next, we investigated FGFR2 protein expression in the subgroup of patients with FGFR2 GAs. At IHC analysis, all the seven cases resulted positive for FGFR2 expression (IHC score ≥ 2 +) (Fig. 3A). Of note, cases T30 and T35 (carrying p.C382R and p.Y375C mutations, respectively) showed FGFR2 positivity only in the cytoplasm of tumor cells, whereas in the remaining five cases FGFR2 positivity was observed both in the cytoplasm and cell membrane (Supplementary Fig. 5). Further Western blotting analysis showed that T30 and T35 cases expressed only the unglycosylated/partially glycosylated form of FGFR2 protein (lower band from ~ 75 to ~ 100 KDa), whereas the other cases also expressed the fully glycosylated form (upper band ~ 150KDa) (Fig. 3B). This finding is in line with previous studies showing that glycosylation is critical for proper FGFR2 protein trafficking to cell membrane, and that unglycosylated/partially glycosylated forms of the receptor do not reach the cell surface^30^.

In order to evaluate the occurrence of activated FGFR2 signalling in the tumor tissue of iCCA patients with FGFR2 GAs, we assessed the levels of protein phosphorylation of both FGFR2 and FRS2α, its main downstream target. Because FGFR2 p.F276C, p.C382R and p.Y375C have been previously shown to be activating mutations^17,31^, cases T5, T30 and T35 were chosen as positive controls for activated FGFR2 signalling in our study population, whereas the human non-malignant cholangiocyte H69 cell line as negative control. As shown in Fig. 3A,B, a high expression of phospho-FGFR2 and phospho-FRS2α protein was found in the iCCA cases with *FGFR2* GAs by IHC and Western blotting analyses, confirming constitutive activation of FGFR2 signalling in this subgroup of patients.

### FGFR2 expression and signalling activation in iCCA naïve patients with wild-type FGFR2

The levels of FGFR2 protein expression and signalling activation were then assessed in the subgroup of iCCA patients with wild-type *FGFR2*. Among these patients, 8 out of 29 cases (27.6%) showed a high FGFR2 expression, 13 cases (44.8%) a moderate expression, 6 cases (20.7%) a weak positivity and 2 cases (6.9%) did not express FGFR2 protein in the tumor tissue (Supplementary Fig. 6a–d). Overall, 21 cases with wild-type *FGFR2* (72.4%) resulted positive for FGFR2 expression (IHC score ≥ 2 +); the remaining 8 cases (27.6%) resulted negative (IHC score ≤ 1 +) and were therefore excluded from further molecular analysis.

**Figure 4:**
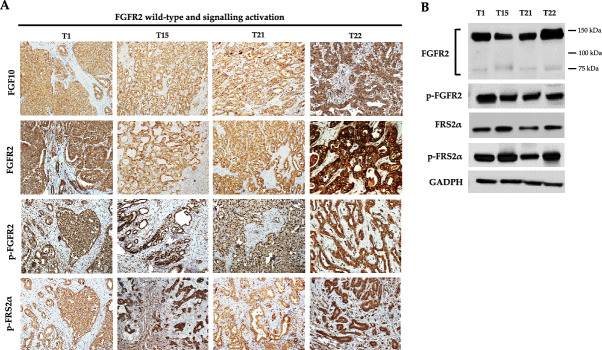
(**A**) Representative IHC images of FGF10 ligand, FGFR2, phospho-FGFR2 and phospho-FRS2α protein expression in 4 iCCA naïve patients with FGFR2 wild-type and positive for activated FGFR2 signalling in the tumor tissue. Magnification 20X; (**B**) Western blotting analysis of FGFR2, phospho-FGFR2, phospho-FRS2α and FRS2α protein expression in iCCA naïve patients with FGFR2 wild-type and positive for activated FGFR2 signalling in the tumor tissue. In FGFR2 immunoblotting, the lower band from ~ 75 to ~ 100 KDa refers to the unglycosylated/partially glycosylated form of FGFR2 protein, whereas the upper band of ~ 150KDa to the fully glycosylated form of the receptor. GAPDH was used as a quantitative control for equal loading.

Interestingly, among the 21 FGFR2-positive cases, we identified two distinct subgroups of patients on the basis of FGFR2 signalling activation in the tumor tissue. The first subgroup of patients included 4 cases (19.0%) that were classified as “positive for FGFR2 signalling activation”, as they showed high expression of both phosphorylated FGFR2 and phosphorylated FRS2α (Fig. 4A,B); these 4 cases (T1, T15, T21 and T22) also showed a high expression of FGF10 protein, the main FGFR2 ligand (Fig. 4A).

**Figure 5:**
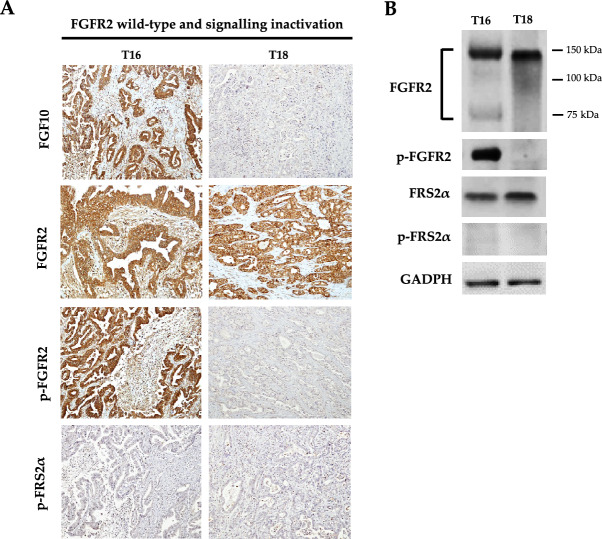
(**A**) Representative IHC images of FGF10 ligand, FGFR2, phospho-FGFR2 and phospho-FRS2α protein expression in iCCA naïve patients with FGFR2 wild-type and negative for activated FGFR2 signalling in the tumor tissue. T16 was chosen as a representative case of patients expressing FGF10, FGFR2 and phospho-FGFR2, but not phospho-FRS2α protein; T18 was chosen as a representative case of patients expressing FGFR2 protein, but negative for FGF10, phospho-FGFR2 and phospho-FRS2α expression. Magnification 20X; (**B**) Western blotting analysis of FGFR2, hosphor-FGFR2, hosphor-FRS2α and FRS2α protein expression in iCCA naïve patients with FGFR2 wild-type and negative for activated FGFR2 signalling in the tumor tissue. In FGFR2 immunoblotting, the lower band from ~ 75 to ~ 100 Kda refers to the unglycosylated/partially glycosylated form of FGFR2 protein, whereas the upper band of ~ 150Kda to the fully glycosylated form of the receptor. GAPDH was used as a quantitative control for equal loading.

The second subgroup of patients included 17 cases (81.0%) classified as “negative for FGFR2 signalling activation”. Among these patients, 10 out 17 cases expressed FGF10 ligand, FGFR2 and phosphorylated FGFR2 in the tumor tissue, but not phosphorylated FRS2α protein, the main downstream effector of activated FGFR2 signalling (Fig. 5A,B); the other seven cases expressed wild-type FGFR2 protein, but were negative for FGF10 ligand, phosphorylated FGFR2 and phosphorylated FRS2α protein expression (Fig. 5A,B).

Overall, these findings unravelled that, in the cohort of patients expressing wild-type FGFR2 protein, only a subgroup (19.0%) of cases showed a concomitant activation of FGFR2 signalling in the tumor tissue; in the remaining cases, despite the expression of the receptor, the downstream signalling cascade resulted inactivated.

## Discussion

The increasing knowledge of iCCA genomic landscape is expected to pave the way for the development of more effective treatments for this dismal disease in the near future. Recently, *FGFR2* fusions/rearrangements have emerged as one of the most promising therapeutic targets in iCCA and the portfolio of available FGFR inhibitors is rapidly increasing^32^. The unprecedented efficacy and good tolerability showed by FGFR inhibitors in clinical trials have fostered the approval of pemigatinib, infigratinib and futibatinib as second-line treatments in locally advanced or metastatic iCCA patients with *FGFR2* fusion or rearrangements^9–11^. The efficacy of FGFR inhibitors in iCCA patients with *FGFR2* fusions/rearrangements is under investigation also in first-line setting (FIGHT-302, PROOF 301 and FOENIX-CCA3 trials), which has the potential to further shape the scenario of front-line treatment for this disease. Despite the growing interest in the use of FGFR inhibitors in different lines of treatment and in different settings of iCCA patients, some important issues still remain to be addressed, namely, the identification of effective predictive biomarkers of response to these drugs, and a better understanding of the molecular mechanisms driving primary and acquired resistance.

Currently the selection of iCCA patients for FGFR targeted therapies is based on the detection of *FGFR2* GAs in the tumor tissue by genetic testing. However, clinical practice has clearly shown that such detection does not always represent a reliable predictive biomarker of response, as only a subgroup of patients with *FGFR* GAs benefits from these drugs (ORR:14.8% for infigratinib; ORR: 35.5% for pemigatinib; ORR: 43% for futibatinib)^11,33,34^. Both reversible and irreversible FGFR inhibitors are able to block FGFR kinase activity by their binding to the ATP pocket in the TKD of the receptor, that in turn results in a strong inhibition of FGFR-mediated oncogenic pathways^13^. This mechanism of action suggests that not only the status of activation of the receptor, but also the status of activation of the downstream FGFR signalling cascade may play a central role in achieving a response in patients receiving these drugs.

To our knowledge this represents the first study performing concomitant molecular characterization of *FGFR2* status and FGFR2 signalling activation in the tumor tissue of iCCA naïve patients. The first interesting finding emerging from our analysis refers to patients expressing wild-type *FGFR2*. Indeed, within this group of patients, we uncovered two distinct subgroups classified as positive and negative for FGFR2 signalling activation. Patients classified as “positive” expressed all the components of activated FGFR2 signalling, including the FGF10 ligand, phosphorylated FGFR2 and phosphorylated FRS2α proteins. Conversely patients classified as “negative”, despite the expression of some of these components, lacked phosphorylated FRS2α protein expression, the main downstream target of activated FGFR2.

Despite the small sample size of the iCCA cases analysed in this study, the identification of two distinct subgroups within FGFR2 wild-type patients could have significant clinical implications for selection of patients eligible for the treatment with FGFR inhibitors. Indeed, it is conceivable that also the subgroup of FGFR2 wild-type patients with activated FGFR signalling, currently excluded to FGFR targeted therapies, may benefit from inhibition of FGFR kinase activity by these drugs.

Unfortunately, as our cohort of patients did not receive any treatment with FGFR inhibitors after surgery, we were unable to address this issue in the present study. Interestingly, a recent in vitro study reported that the FGFR inhibitor infigratnib was able to inhibit FGF10/FGFR2 signalling in CCA cell lines, resulting in suppression of cell migration^35^. Moreover, a partial response to the FGFR inhibitor rogaratinib has been observed in 15% of cancer patients overexpressing *FGFR* mRNA; of these, 67% of cases were negative for *FGFR* GAs^36^. This suggests, in line with our hypothesis, the existence of a subgroup of FGFR wild-type cancer patients that is likely to respond to FGFR inhibitors besides the subgroup of patients with *FGFR* GAs.

A second interesting finding emerged from the present study is the occurrence of targetable FGFR2 p.F276C, p.C382R and p.Y375C mutations in 8.3% (3/36) of iCCA naïve patients. Notably, such mutations have been reported in a previous study on iCCA, although with a lower frequency (2.2% of cases) and in different settings of patients^37^. FGFR2 p.C382R and p.Y375C mutations have been also detected in the FIGHT-202 trial, with the former representing the most common variant occurring in iCCA patients without *FGFR2* fusions or rearrangements^22^. More recently, a retrospective analysis of 6,130 iCCA patients from the FoundationCORE database reported that FGFR2 p.C382R, p. F276C and p.Y375C point mutations are common molecular events in iCCA, occurring in 22.7%, 22% and 19.9% of cases, respectively^15^. Although these studies did not clearly specify whether tumor sampling and sequencing analysis were performed before any systemic treatment, overall these findings suggest that F276, Y375 and C382 sites of FGFR2 receptor likely represent mutational hot-spots in iCCA. The clinical actionability of these FGFR2 mutations has been recently investigated in some studies. The first case refers to an iCCA patient who became refractory to prior chemotherapy regimens. At disease progression, the FGFR2 p.F276C mutation was detected by tumor sequencing and the patient was treated with infigratinib, achieving a partial response after 2 months of treatment and maintaining a response for additional 4 months; afterwards new lesions developed and the treatment was discontinued^31^. In the FIGHT-202 study, 3 out of 4 patients carrying the FGFR2 p.C382R mutation achieved a stable disease during treatment with pemigatinib, with a median progression-free survival (PFS) ranging from 4.0 to 9.0 months^11^. More recently, complete functional remission was reported after treatment with pemigatinib in an iCCA patient carrying the FGFR2 p.C382R mutation. The patient received prior treatment with gemcitabine, cisplatin and nab-paclitaxel and, at progression, FGFR2 p.C382R mutation was detected in the tumor tissue and blood sample by sequencing analysis^37,38^. Furthermore, a phase I dose-expansion trial on patients with advanced solid tumors showed that futibatinib was active against a broad spectrum of tumors harbouring *FGFR* GAs; as for iCCA, although patients with *FGFR2* fusions/rearrangements experienced the most clinical benefit (ORR, 25.4%), an objective response was also observed in two patients carrying the FGFR2 p.W290C and p.C382R mutations^39^. Overall, these findings suggest the clinical actionability of the FGFR2 missense mutations detected in our cohort of iCCA naïve patients. In this scenario, the efficacy of systemic chemotherapy plus FGFR inhibitors may deserve future clinical investigations in this setting of patients. An important issue that remains to be clarified, however, is whether chemotherapy may lead to a selection of tumor cell subclones with *FGFR2* GAs, a molecular event that may confer resistance to the chemotherapy itself. As stated above, the occurrence of FGFR2 p.F276C and p.C382R mutations was detected in two iCCA patients treated with prior chemotherapy schedules^31,38^. Unfortunately, as in both cases tumor sequencing was performed only at disease progression, it is not possible to establish whether these mutations were already present at tumor diagnosis or were selected following chemotherapy treatments. Prospective studies evaluating the occurrence of *FGFR2* GAs ab initio and during treatment in patients receiving chemotherapy are therefore warranted to address this issue, as they could open the way for chemotherapy in combination with FGFR inhibitors in selected iCCA patients.

**Figure 6:**
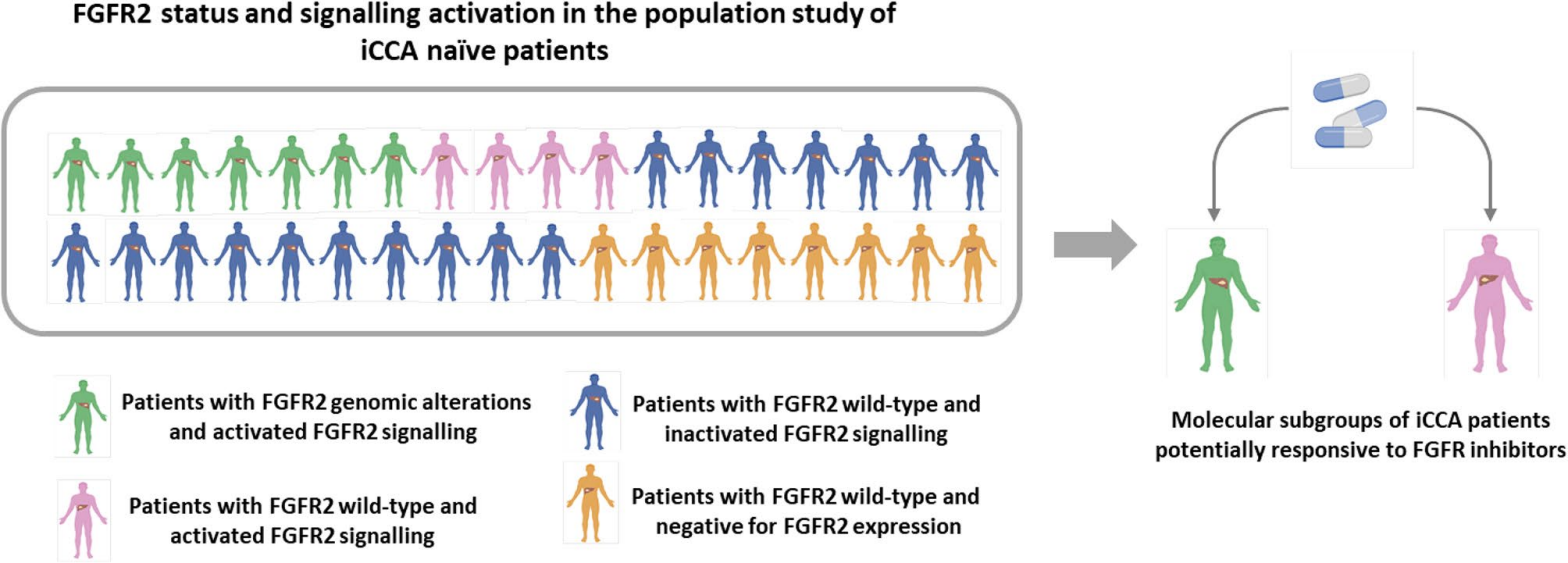
Schematic representation of FGFR2 status and signalling activation in the thirty-six iCCA naïve patients included in the study.

In summary, here we showed that activation of FGFR2 signalling is a relatively frequent event in iCCA naïve patients, either in those with *FGFR2* GAs, or in a subgroup of patients with FGFR2 wild-type (Fig. 6). This finding could entail that even patients without *FGFR2* GAs, but expressing activated FGFR2 signalling, could benefit from FGFR inhibitors. Accordingly, concomitant molecular assessment of phosphorylated FGFR2 and phosphorylated FRS2α protein expression in tumor tissue by routine methodologies such as IHC analysis could represent a more reliable biomarker, rather than the only genetic testing of FGFR status, to select iCCA patients candidate to FGFR targeted therapies. This molecular screening could avoid the prescription of these drugs to patients (including those with FGFR2 GAs) that are unlikely to respond to these treatments because of inactivated FGFR signalling in tumor tissue, with a significant reduction in terms of clinical and financial toxicities. Moreover, it is worth to underline that, when selecting iCCA patients eligible to FGFR targeted therapies, also co-occurrence of other driver mutations should be carefully considered, as they could significantly impact treatment response, as reported in other cancer types^40,41^. Previous molecular studies suggest that co-mutational events are not completely random in iCCA, as some of them tend to co-occur with a higher frequency than others^15^. In particular, FGFR2 alterations have been reported to be enriched in iCCAs with BAP1 mutations; conversely, co-alterations in ARID1A, PBRM1 and TP53 genes seem to be less common in iCCA patients with FGFR2 GAs compared to those ones with FGFR2 wild-type^15^. As to IDH mutations, they have been shown to be mutually exclusive with FGFR2 genomic rearrangements, but to occur in 11% of patients with FGFR2 point mutations^15^. In line with this last finding, among the seven iCCA patients with FGFR2 GAs of the present study, we detected one case carrying concomitant FGFR2 p.F276C and IDH1 p.R132C mutations. In the four patients with FGFR2 wild-type and activated FGFR2 signalling, one case was found to harbour the IDH1 p.R132C mutation, while another the IDH2 p.R172G actionable mutation (with level of evidence 3B). Overall these findings suggest that, in molecularly selected subgroups of iCCA patients, combined therapeutic strategies with FGFR inhibitors and drugs targeting oncogenic drivers in iCCA may be required to achieve a treatment response and clinical benefit.

We strongly encourage future clinical studies to confirm our preliminary findings, as they could widen the proportion of iCCA patients eligible for treatment with FGFR inhibitors, alone or in combination, outside currently approved indications.

### Supplementary Information

Supplementary Legends.

Supplementary Figure 1.

Supplementary Figure 2.

Supplementary Figure 3.

Supplementary Figure 4.

Supplementary Figure 5.

Supplementary Figure 6.

Supplementary Figure 7.

Supplementary Table 1.

## Data Availability

All data generated or analysed during this study are included in this article and its supplementary material files. Further enquiries can be directed to the corresponding author.

## Abbreviations

iCCA: Intrahepatic cholangiocarcinoma
FGFR: Fibroblast growth factor receptor
FRS2α: FGFR substrate 2α
IDH: Isocitrate dehydrogenase
MAPK: Mitogen activated protein kinase
PI3K: Phosphoinositide-3-kinase
GAs: Genomic alterations
FDA: Food and Drug Administration
ECD: Extracellular domain
TD: Transmembrane domain
TKD: Tyrosine kinase domain
WES: Whole exome sequencing analysis
IHC: Immunohistochemistry
FISH: Fluorescence in situ hybridization
MSA: Multiple sequence alignment
MMR: Mismatch repair
MLH1: MutL homolog 1
PMS2: Mismatch repair endonuclease 2
MSH1: MutS homolog 1
MSH6: MutS homolog 6
GAPDH: Glyceraldehyde-3-phosphate dehydrogenase
