# Supplementary material for: Activated FGFR2 signalling as a biomarker for selection of intrahepatic cholangiocarcinoma patients candidate to FGFR targeted therapies
Source: Sci Rep. 2024 Feb 7;14:3136. doi: 10.1038/s41598-024-52991-8 (PMC10850506; doi:10.1038/s41598-024-52991-8)
Supplement: Supplementary file 1 — Supplementary Legends. [file 41598_2024_52991_MOESM1_ESM.docx]

**LEGEND TO SUPPLEMENTARY FIGURES AND TABLES**

**Supplementary Figure 1:** Total number of somatic coding variants for tumor (upper panel) and mutational landscape of the thirty-six iCCA naïve patients included in the study. Mutated genes are listed on the right-side and each column represents a patient. Green: missense mutations; purple: splice-site mutations; red: non-sense mutations; yellow: non-frameshift mutations; orange: frameshift mutations.

**Supplementary Figure 2**: Expression pattern of MMR proteins in the tumor tissue of three hypermutated iCCA naïve patients. Cases 12 and 18 showed the loss of MLH1 and PMS2 protein expression, whereas case 18 the loss of MSH2 and MSH6 protein expression (magnification 20X).

**Supplementary Figure 3: A)** Schematic representation of the intragenic location of FGFR2 p.F276C, p.Y375C and p.C382R missense mutations; **B)** MSA for FGFR2 protein showing highly conserved F276, Y375 and C382 residues among six different species. On the left, UniProt accession numbers of each protein are shown. Human: Homo sapiens; PANTR: Pan troglodytes (Chimpanzee); BOVINE: Bos Taurus; MOUSE: Mus musculus; RAT: Rattus norvegicus; ZEBRAFISH: Danio rerio.

**Supplementary Figure 4:** FISH analysis with FGFR2/Chromosome Control 10 probe showing a gain of chromosome 10 in T3, T9, T11 and T5 cases. DAPI counterstain; Magnification 60X.

**Supplementary Figure 5:** FGFR2 subcellular localization in iCCA naïve patients with FGFR2 GAs. Red arrows show FGFR2 membrane localization in cases T5, T13, T3, T9 and T11. Magnification 40X.

**Supplementary Figure 6:** Representative IHC images for high (a), moderate (b), weak (c) and negative (d) FGFR2 protein expression in iCCA naïve patients with FGFR2 wild-type. See “Material and methods section” for score definition. Magnification 20X.

**Supplementary Table1:** List of actionable mutations in the thirty-six iCCA naïve patients included in the study ordered according to OncoKB level of evidence.
