# Supplementary figures and images for: Activated FGFR2 signalling as a biomarker for selection of intrahepatic cholangiocarcinoma patients candidate to FGFR targeted therapies
Source: Sci Rep. 2024 Feb 7;14:3136. doi: 10.1038/s41598-024-52991-8 (PMC10850506; doi:10.1038/s41598-024-52991-8)

## Slide 1
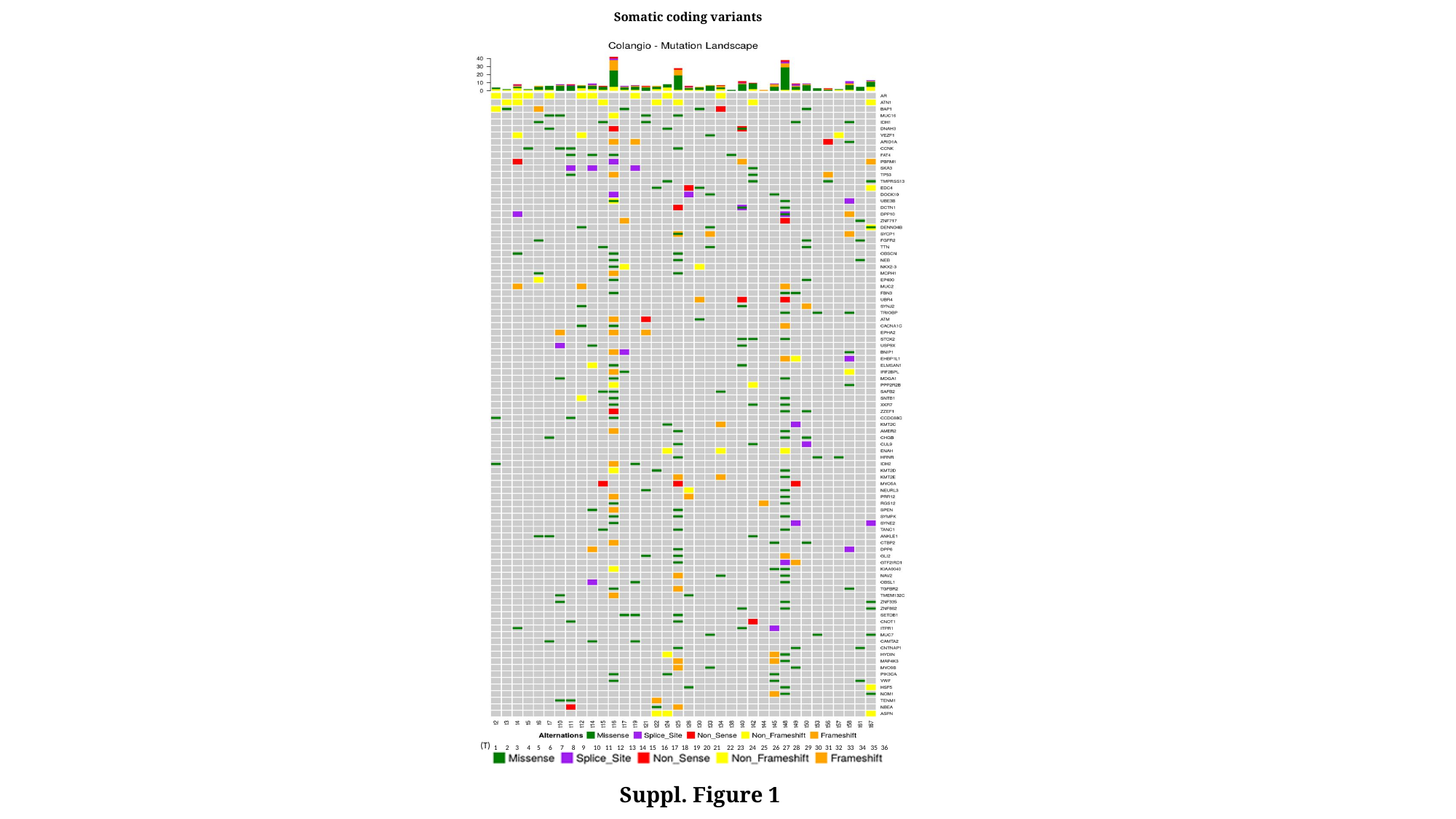

Somatic coding variants
Suppl. Figure 1
(T)
1 2 3 4 5 6 7 8 9 10 11 12 13 14 15 16 17 18 19 20 21 22 23 24 25 26 27 28 29 30 31 32 33 34 35 36

Supplement: Supplementary file 2 — Supplementary Figure 1. [file 41598_2024_52991_MOESM2_ESM.pptx]

## Slide 1
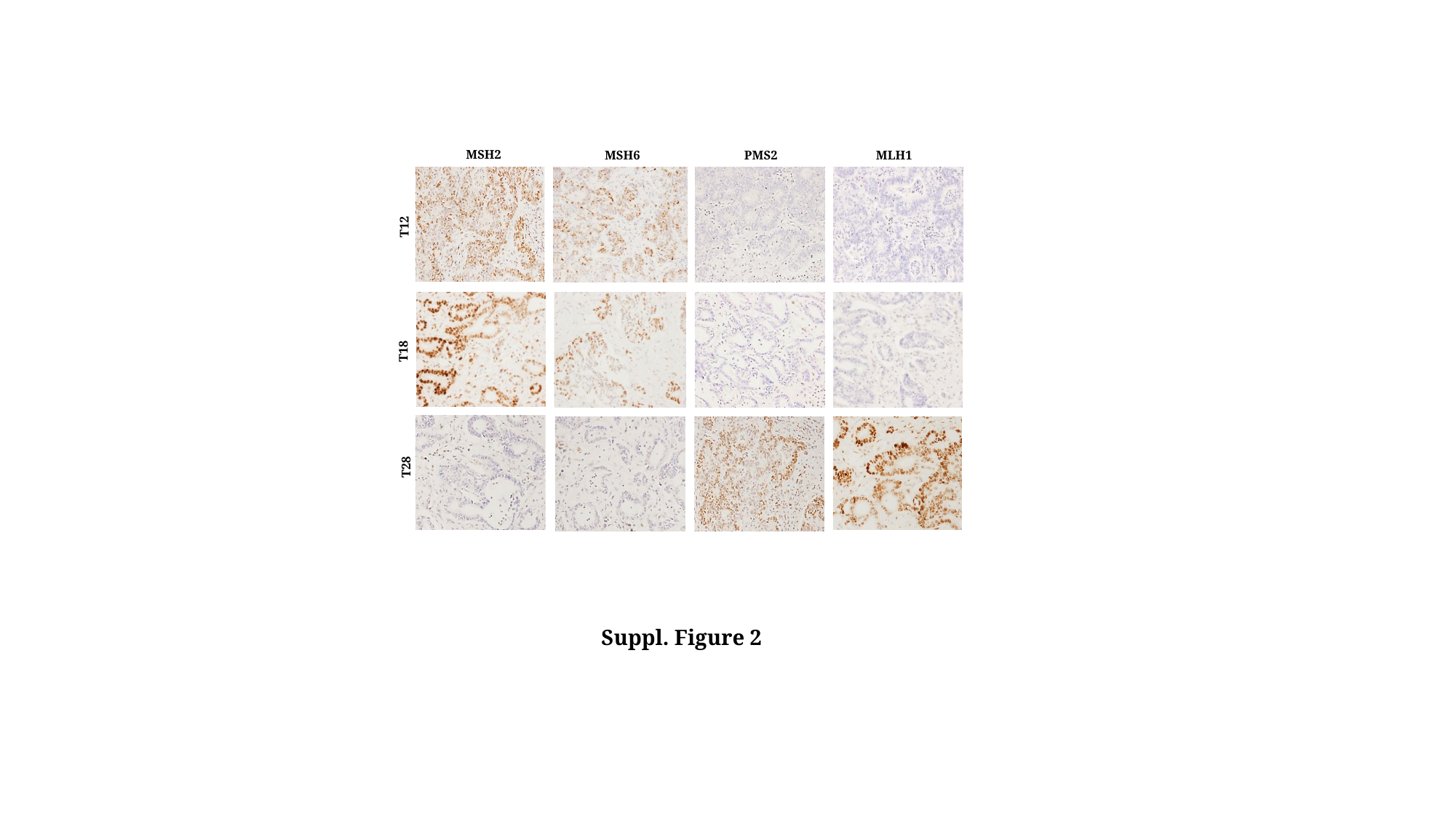

MSH2
MSH6
PMS2
MLH1
 T12
T18
T28
Suppl. Figure 2

Supplement: Supplementary file 3 — Supplementary Figure 2. [file 41598_2024_52991_MOESM3_ESM.pptx]

## Slide 1
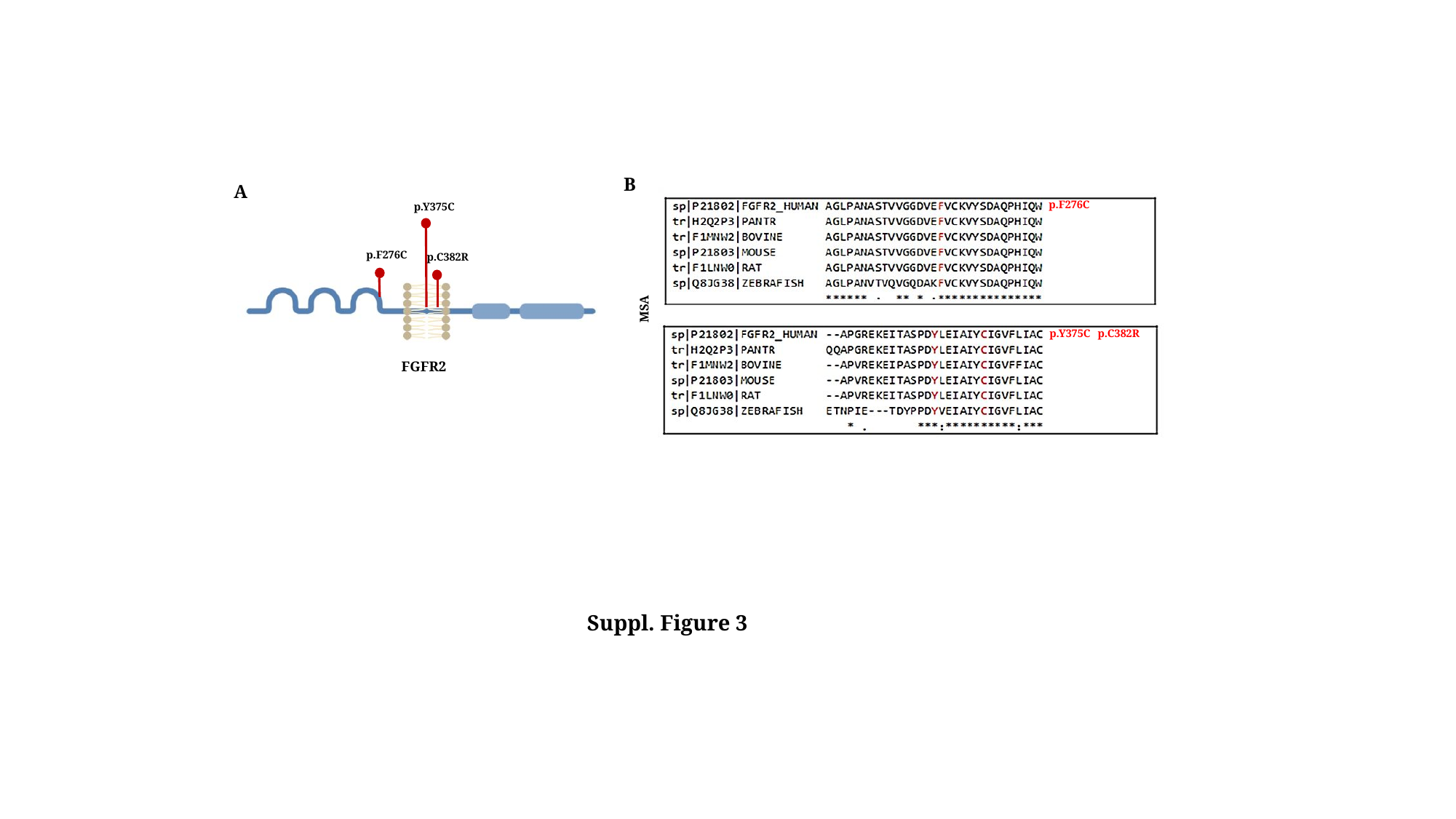

B
p.F276C
p.Y375C
p.C382R
MSA
A
p.Y375C
p.F276C
p.C382R
FGFR2
Suppl. Figure 3

Supplement: Supplementary file 4 — Supplementary Figure 3. [file 41598_2024_52991_MOESM4_ESM.pptx]

## Slide 1
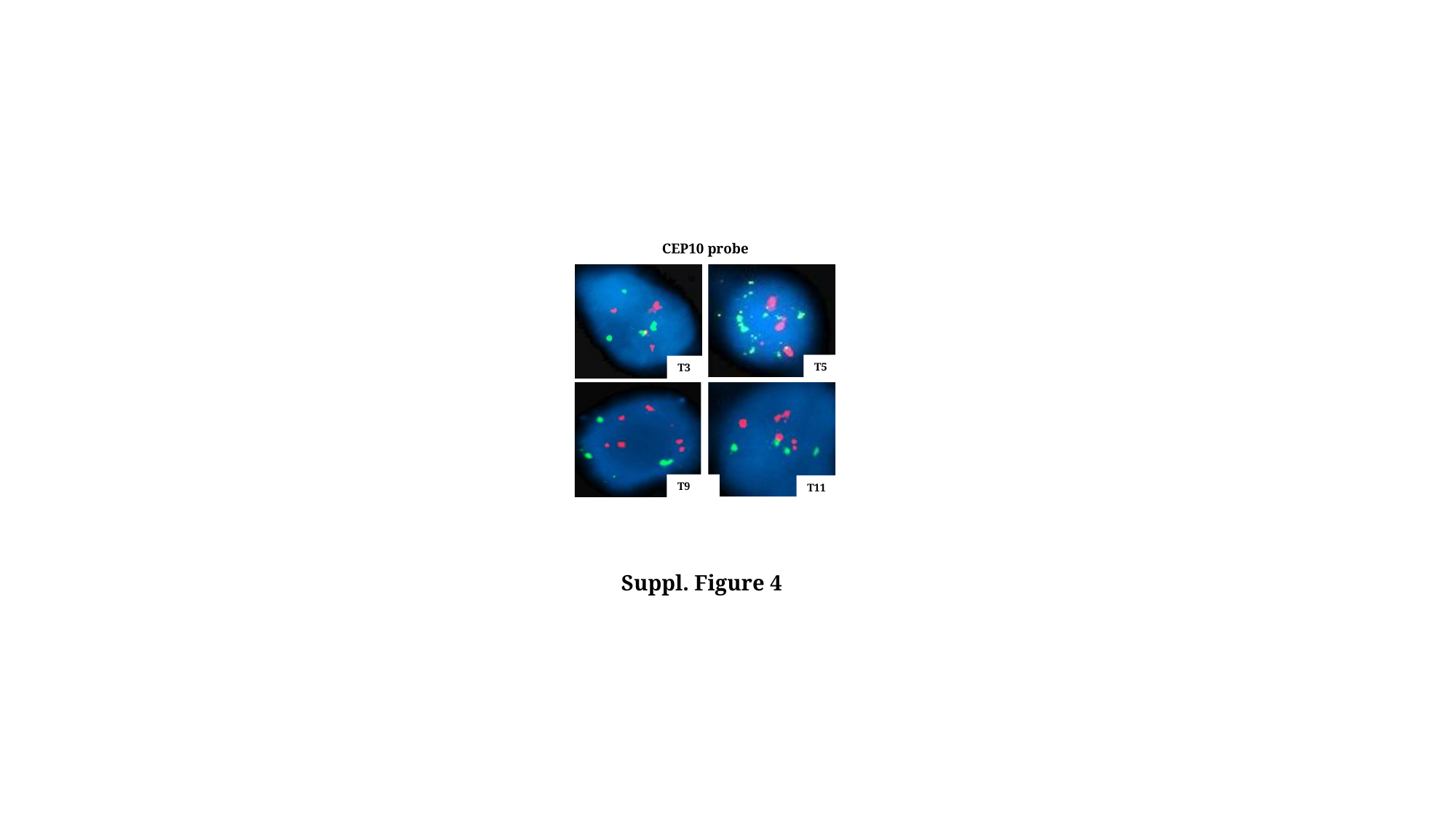

CEP10 probe
T5
T3
T11
T9
Suppl. Figure 4

Supplement: Supplementary file 5 — Supplementary Figure 4. [file 41598_2024_52991_MOESM5_ESM.pptx]

## Slide 1
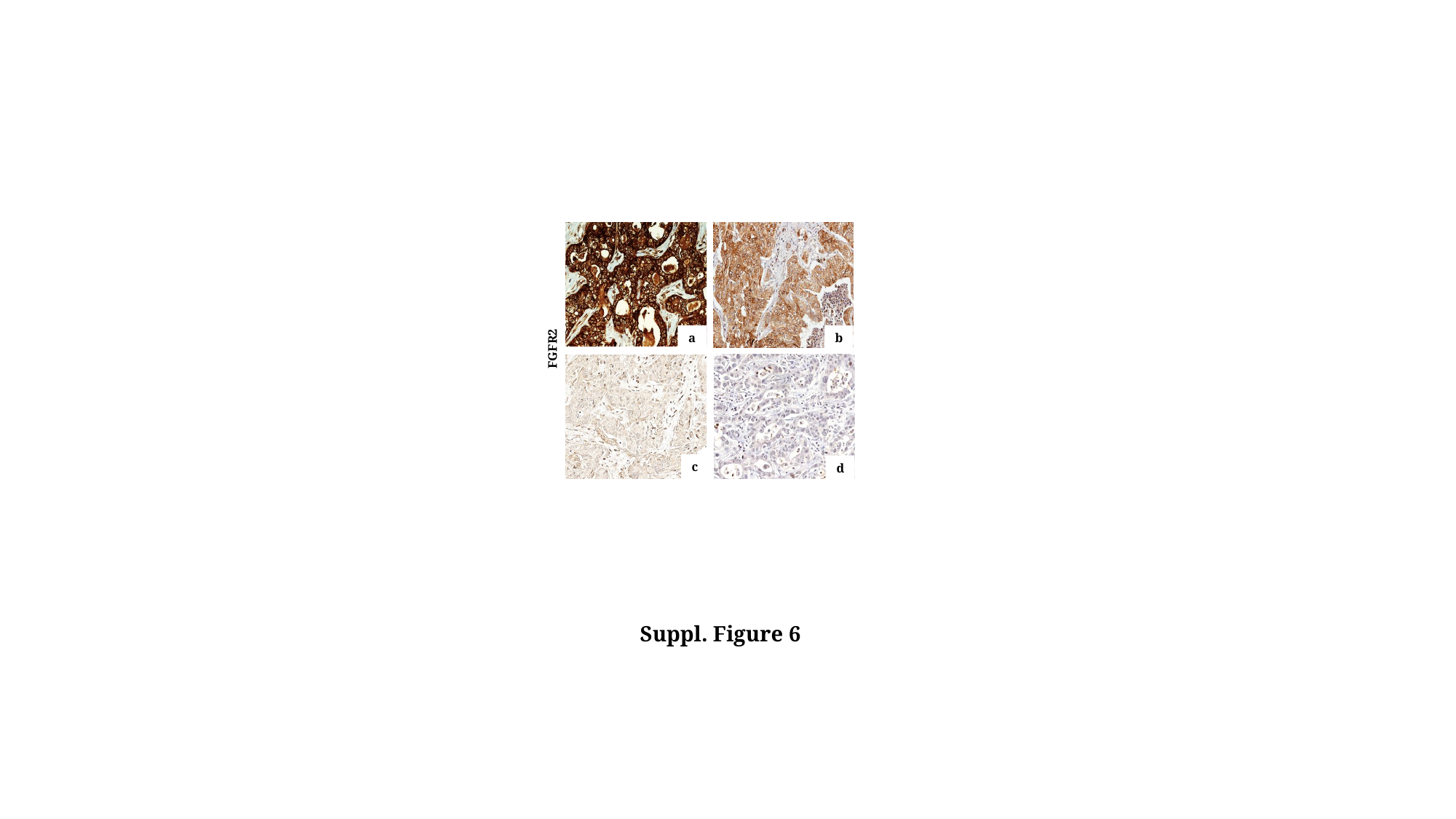

a
b
FGFR2
c
d
Suppl. Figure 6

Supplement: Supplementary file 7 — Supplementary Figure 6. [file 41598_2024_52991_MOESM7_ESM.pptx]
