# Supplementary material for: Activated FGFR2 signalling as a biomarker for selection of intrahepatic cholangiocarcinoma patients candidate to FGFR targeted therapies
Source: Sci Rep. 2024 Feb 7;14:3136. doi: 10.1038/s41598-024-52991-8 (PMC10850506; doi:10.1038/s41598-024-52991-8)
Supplement: Supplementary file 6 — Supplementary Figure 5. [file 41598_2024_52991_MOESM6_ESM.pptx]

## Slide 1
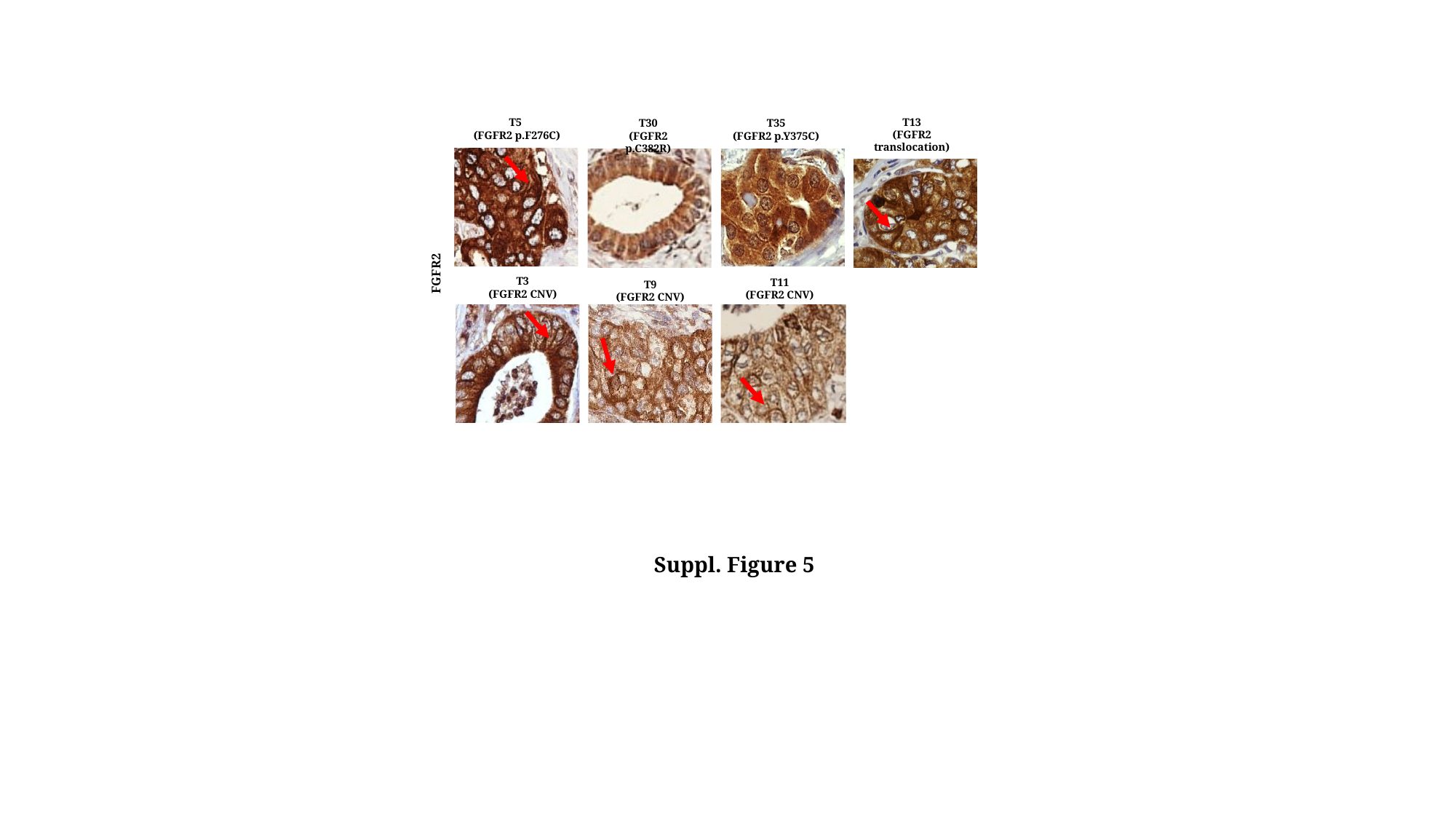

T13
(FGFR2 translocation)
T5
(FGFR2 p.F276C)
T30
(FGFR2 p.C382R)
T35
(FGFR2 p.Y375C)
FGFR2
T3
(FGFR2 CNV)
T11
(FGFR2 CNV)
T9
(FGFR2 CNV)
Suppl. Figure 5
