# Supplementary material for: Activated FGFR2 signalling as a biomarker for selection of intrahepatic cholangiocarcinoma patients candidate to FGFR targeted therapies
Source: Sci Rep. 2024 Feb 7;14:3136. doi: 10.1038/s41598-024-52991-8 (PMC10850506; doi:10.1038/s41598-024-52991-8)
Supplement: Supplementary file 8 — Supplementary Figure 7. [file 41598_2024_52991_MOESM8_ESM.pptx]

## Slide 1
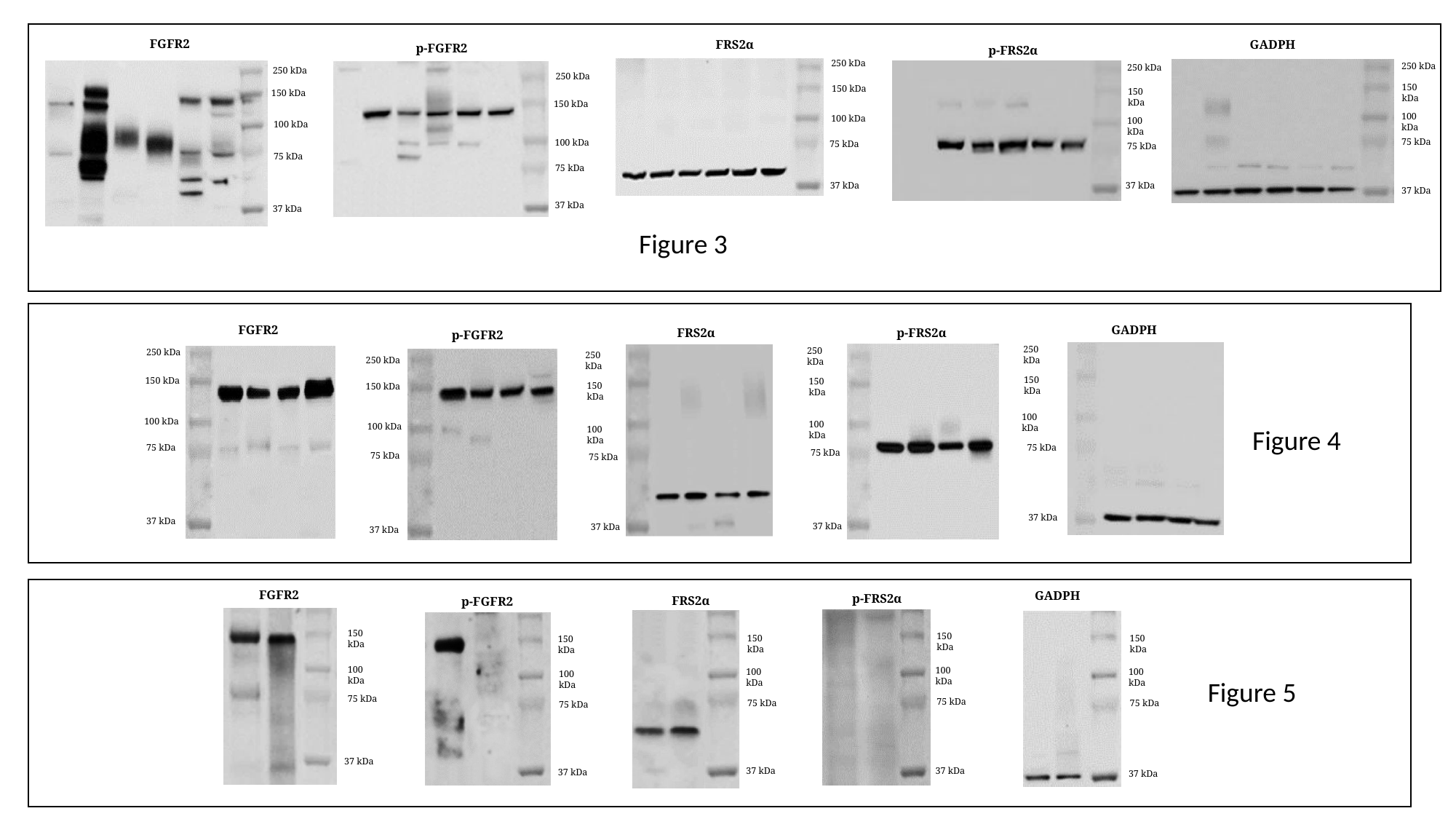

FGFR2
150 kDa
100 kDa
75 kDa
37 kDa
250 kDa
FRS2α
250 kDa
150 kDa
100 kDa
75 kDa
GADPH
p-FGFR2
150 kDa
100 kDa
75 kDa
250 kDa
p-FRS2α
150 kDa
100 kDa
75 kDa
250 kDa
250 kDa
150 kDa
100 kDa
75 kDa
37 kDa
37 kDa
37 kDa
37 kDa
Figure 3
FGFR2
250 kDa
150 kDa
100 kDa
75 kDa
37 kDa
GADPH
250 kDa
150 kDa
100 kDa
75 kDa
37 kDa
FRS2α
250 kDa
150 kDa
100 kDa
75 kDa
37 kDa
p-FRS2α
250 kDa
150 kDa
100 kDa
75 kDa
37 kDa
p-FGFR2
250 kDa
150 kDa
100 kDa
75 kDa
37 kDa
Figure 4
FGFR2
150 kDa
100 kDa
75 kDa
37 kDa
GADPH
150 kDa
100 kDa
75 kDa
37 kDa
p-FRS2α
150 kDa
100 kDa
75 kDa
37 kDa
FRS2α
150 kDa
100 kDa
75 kDa
37 kDa
p-FGFR2
150 kDa
100 kDa
75 kDa
37 kDa
Figure 5
