# Supplementary material for: Activated FGFR2 signalling as a biomarker for selection of intrahepatic cholangiocarcinoma patients candidate to FGFR targeted therapies
Source: Sci Rep. 2024 Feb 7;14:3136. doi: 10.1038/s41598-024-52991-8 (PMC10850506; doi:10.1038/s41598-024-52991-8)
Supplement: Supplementary file 9 — Supplementary Table 1. [file 41598_2024_52991_MOESM9_ESM.docx]

**Supplementary Table 1**

| **Sample** | **Gene** | **Protein** | **OncoKB level of evidence** |
| --- | --- | --- | --- |
| T5 | IDH1 | p.R132C | 1 |
| T11 | IDH1 | p.R132C | 1 |
| T15 | IDH1 | p.R132C | 1 |
| T29 | IDH1 | p.R132C | 1 |
| T34 | IDH1 | p.R132C | 1 |
| T1 | IDH2 | p.R172G | 3B |
| T14 | IDH2 | p.R172G | 3B |
| T12 | PIK3CA | p.G1049R | 3B |
| T17 | PIK3CA | p.Q546R | 3B |
| T12 | ATM | p.A1812fs | 3B |
| T18 | PTCH1 | p.P1051fs | 3B |
| T2 | NRAS | p.G12A | 3B |
| T5 | FGFR2 | p.F276C | 4 |
| T30 | FGFR2 | p.C382R | 4 |
| T35 | FGFR2 | p.Y375C | 4 |
| T3 | KRAS | p.G12V | 4 |
| T16 | KRAS | p.G12D | 4 |
| T21 | NF1 | p.K1290fs | 4 |
